# Neurogenesis of the scallop *Azumapecten farreri*: from the first larval sensory neurons to the definitive nervous system of juveniles

**DOI:** 10.1186/s12983-022-00468-7

**Published:** 2022-08-03

**Authors:** Marina Kniazkina, Vyacheslav Dyachuk

**Affiliations:** grid.417808.2 0000 0001 1393 1398 A.V. Zhirmunsky National Scientific Center of Marine Biology, Far Eastern Branch, Russian Academy of Sciences, Vladivostok, 690041 Russia

**Keywords:** Bivalve, Ganglia, Neuromorphology, Serotonin, FMRFamide, Larvae, Catecholamines, Neurogenesis

## Abstract

**Background:**

Scallops are among the best-studied bivalve mollusks. However, adult nervous system and neurogenesis studies of scallops are limited. Here, we studied the localization of neurotransmitters (serotonin/5-HT, FMRFamide, catecholamines) in adult ganglia and larvae of *Azumapecten farreri* using histochemical and immunohistochemical methods.

**Results:**

We found peptide FMRFamide in all adult scallop ganglia, whereas 5-HT-like immunoreactive (lir) somata were exclusively detected in the cerebropleural, pedal, and accessory ganglia. Scallop larval neurogenesis starts with the emergence of the 5-HT-lir neurons, which are part of the apical organ (AO) at the early veliger stage. Near the AO, paired anlagen of cerebral ganglion (CG) developed. 5-HT-lir neurites of the CG innervate the velum, ventral, and dorsal parts of the larva at the late veliger stage. Scallop pediveligers possess 5-HT-lir CG, pleural ganglia, and immunopositive signals in the developing enteric nervous system. FMRFamide-lir is first detected in dorsal, ventral, and AO cells of early veligers. Later, FMRFamide-lir extends to the visceral nervous cord, all ganglia, as well as in the enteric nervous system in pediveligers. Catecholaminergic neurons are detected near the larval mouth, in the vellum, and in the stomach in veligers.

**Conclusions:**

We described the distribution of neurotransmitters of the ganglia in adult scallops and the larval neurodevelopment in *A. farreri.* Immunostaining of neurotransmitters showed that the gross anatomy of adult scallop ganglia, in general, is similar to that in other bivalves, but complicated by the complexity of the structure of the ganglia and the appearance of additional ganglia not described in other molluscs. A comparison of larval neuromorphology suggests that 5-HT-lir structures are more conservative than FMRF-lir structures in Bivalvia. Notably, the latter are much more distributed in scallop *A. farreri* larvae than in other studied bivalves.

**Supplementary Information:**

The online version contains supplementary material available at 10.1186/s12983-022-00468-7.

## Background

Among the great taxonomic and ecological diversity of bivalves, scallops, which are morphologically unique bivalve mollusks, possess developed visual systems [1], muscle systems that exhibit muscle conditions (catch state [2–4]), an immune system [5, 6], and a neurohumoral system providing complex physiological and behavioral responses of bivalves [7, 8]. Among all other organ systems of mollusks, the nervous system and its neurotransmitters are of great importance in the regulation of homeostasis in general, and particularly in various physiological processes; such as regulation of locomotor activity [9], contraction of striated and smooth (catch) muscles including the heart [2, 4], regulation of metabolism [10], circulation, feeding, digestion, reproduction, and osmoregulation [11–15]. In addition, the modulation of neurotransmitter expression leads to a change in the rate of larval development [16].

The gross anatomy of the nervous system in adult scallops has been studied previously [17–19]. The central nervous system (CNS) in the scallop *Azumapecten farreri* consists of three paired cerebropleural ganglia (CPG) and fused pedal and visceral (or parietovisceral) ganglia (VG) [17, 20]. Adult scallop CPG, consisting of a cerebral (anterior) lobe and a pleural (posterior) lobe, are located on either side of the pedal ganglion (PG) that lies at the base of the foot and connects only with the CPG but not with the VG [15, 17, 20]. The VG is located on the ventral surface of the striated adductor muscle and is linked with the CPG via cerebropleuro-visceral connectives.

The VG of scallops is larger than that of other bivalves and has a complex structure consisting of lobes that radiate nerves which innervate the mantle, tentacles, eyes, gills, and osphradia [17, 20]. This study uses immunofluorescence and histochemical approaches to document the localization of various neurotransmitters, such as 5-HT, FMRFamide and catecholamines, in adult ganglia and developmental stages of the scallop *A. farreri.*

## Results

### Neuroanatomy of the adult scallop ganglia

#### FMRFamide-, 5-HT-, and acetylated α-tubulin-like immunoreactivity (lir) in CPG and PG of adult *A. farreri*

**Fig. 1:**
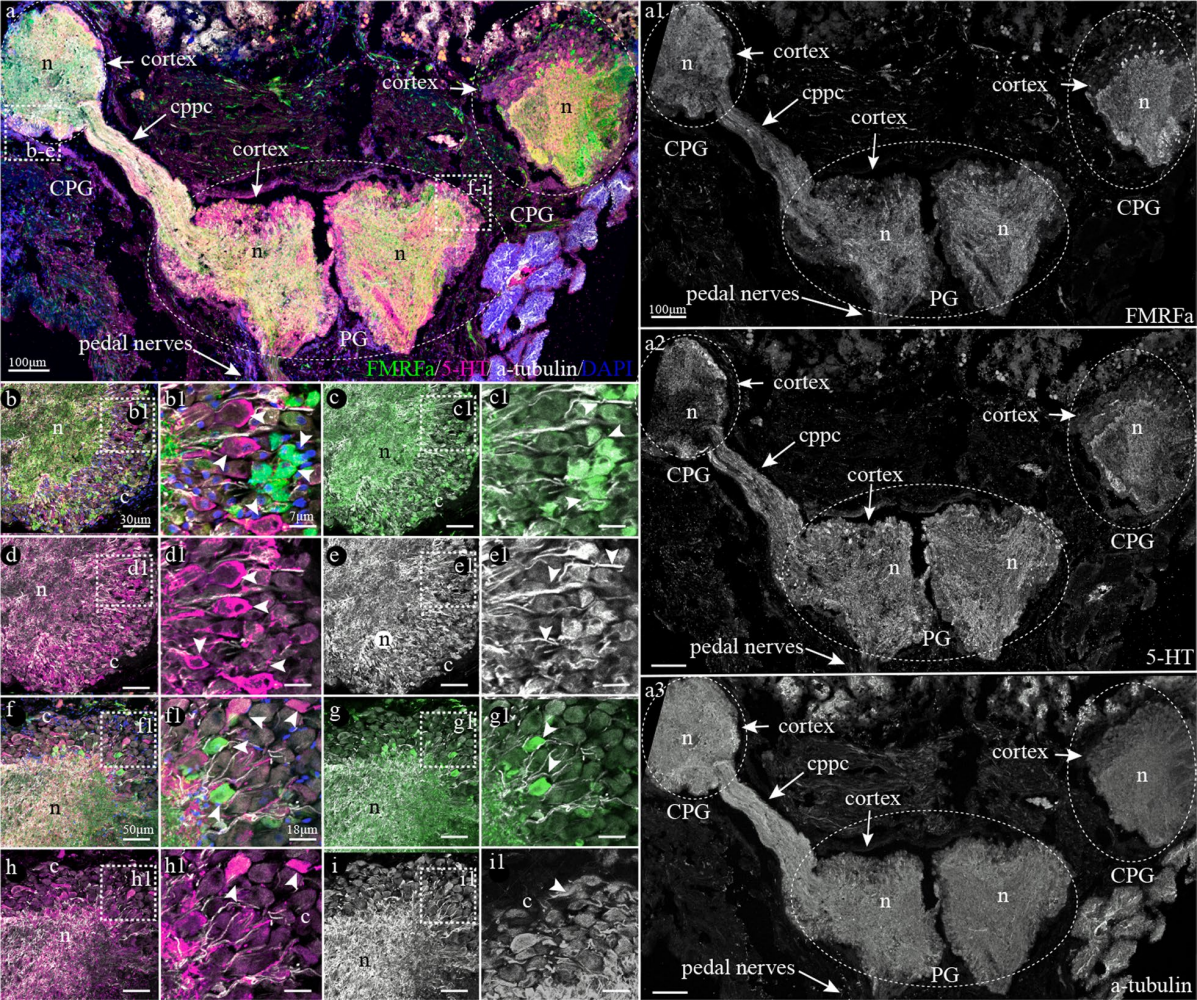
Cryo-section of the cerebropleural (CPG) and pedal (PG) ganglia of the adult scallop *Azumapecten farreri* immunostained with FMRFamide (green), serotonin (magenta), and acetylated α-tubulin (white) antibodies with DAPI (blue). **a** Tissue section view of the CPG connected with PG by cerebropleuro-pedal connective (cppc). The external part of the ganglia includes the neuronal somata (cortex) of ganglia; the inner part of ganglia is intertwined with neurites forming neuropil. Inserts: **b**–**e1** Magnified parts of the CPG. **f**–**i1** The neuropil and cortex of the PG. Scale bars: **a** = 100 μm; **b**, **c**, **d**, **e** = 30 μm; **b1**, **c1**, **d1**, **e1** = 7 μm; **f**, **h**, **g**, **i** = 50 μm; **f1**, **h1**, **g1**, **i1** = 18 μm

We used the terminology accepted by other researchers who have studied this scallop species [17–20]. The nervous system of the adult *A. farreri* consists of paired CPG connected with the PG via cerebropleuropedal connectives (Fig. 1a–a3). Triple immunostaining with FMRFamide, 5-HT, and acetylated α-tubulin antibodies (Fig. 1a) in the neuronal tissue slides showed positive FMRFamide- (Fig. 1a1), 5-HT- (Fig. 1a2), and α-tubulin*-*lir (Fig. 1a3) in both the CPG and PG (Fig. 1a). 5-HT and FMRFamide-lir neurons were detected in the cell body layer (CBL or cortex) of the CPG (Fig. 1b–e1) and PG (Fig. 1f–i1). The immunopositive neurites of the somata are concentrated in the center of the CPG and PG, forming a dense plexus, neuropil (Fig. 1b–i).

FMRFamide- and 5-HT-lir somata did not form dense clusters but were more or less evenly dispersed throughout the outer CBL. Colocalization of acetylated α-tubulin with 5-HT or FMRFamide showed that α-tubulin was expressed in both 5-HT- and FMRFamide-lir neurons of the CPG (Fig. 1b–e1) and PG (Fig. 1f–i).

Thus, FMRFamide and 5-HT antibodies clearly detect neuronal somata of the CPG and PG cortices and their neurites forming the neuropil. Moreover, acetylated α-tubulin was expressed in FMRFamide and 5-HT-lir neurons and neurites.

#### FMRFamide, 5-HT, and acetylated α-tubulin-lir in the VG of adult *A. farreri*

**Fig. 2:**
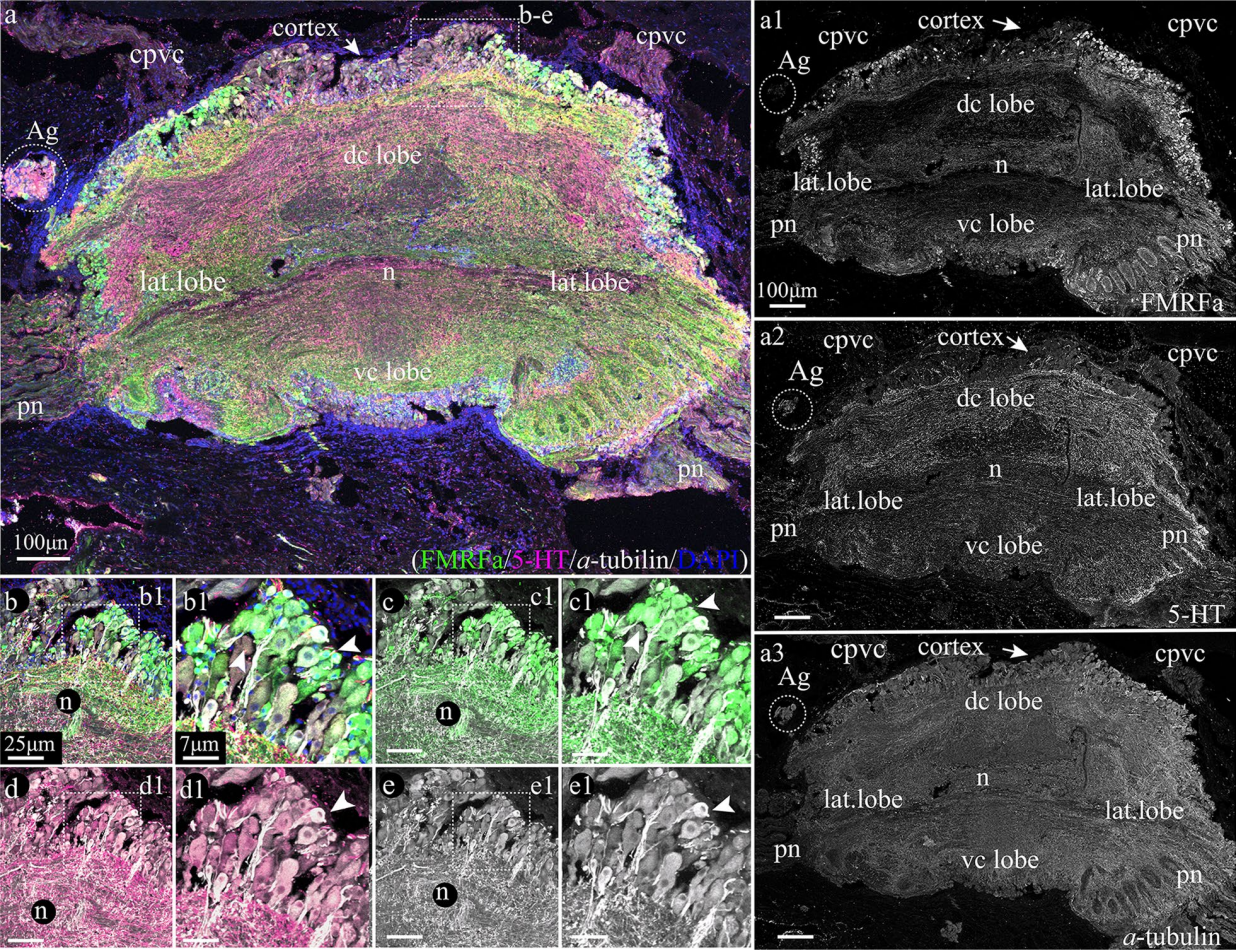
Immunostaining of the visceral ganglion by FMRFamide (green), serotonin (magenta), and acetylated α-tubulin (white) antibodies with DAPI (blue) of the adult scallop *Azumapecten farreri*. **a** Tissue section of the visceral ganglion (VG) with accessory ganglion (Ag)*.* The VG has a dorsal central lobe (dc lobe) ventral central lobe (vc lobe) and two lateral lobes (lat. lobe). Pallial nerves (pn) extending from the lateral lobes. On the upper side from the dorsal central lobe extend cerebropleuro-visceral connective (cpvc). The external part of the ganglia comprising of neuronal cells surrounds the central part of ganglia including the intertwined neurites (neuropil). Inserts: **b**–**e1** Magnified parts of the VG. Scale bars: **a** = 150 μm; **b**, **c**, **d**, **e** = 50 μm; **b1**, **c1**, **d1**, **e1** = 13 μm

The fused VG is the largest and multipart ganglion in adult *A. farreri* (Fig. 2a)*.* VG is linked with CPG via the cerebropleuro-visceral connectives and consists of two dorso-central (anterior), one ventro-central (posterior), and two lateral lobes adjacent to the VG (Fig. 2a). In addition, two accessory ganglia are located adjacent to the VG (Fig. 2a). Immunostaining with FMRFamide, 5-HT, and acetylated α-tubulin antibodies showed that FMRFamide and α-tubulin-lir neurons were found in the CBL and neuropil of VG (Fig. 2a–e1). Although we did not detect 5-HT-lir somata in the CBL of the VG, 5-HT-lir neurites are present in its neuropil (Fig. 2a–e1). As for the CPG and PG, FMRFamide and α-tubulin-lir neurons did not have individual clusters in the VG. However, accessory ganglia mainly consisted of 5-HT-lir neurons with solitary FMRFamide-lir neurons on the periphery of the ganglion (2–3 neurons per tissue section) (Fig. 2a–a2). High-magnification images of immunostaining confirmed the absence of 5-HT-lir neurons in the CBL of the VG, but the presence of 5-HT-lir cell processes together with FMRFamide-lir fibers, which formed the neuropil (Fig. 2b–e1).

Thus, scattered FMRFamide-lir neurons were found throughout all ganglia, while 5-HT appears to be the dominant neurotransmitter in the accessory ganglia but was absent from the CBL of the VG.

### Neurodevelopment

#### Larval stages of the scallop *A. farreri*

**Fig. 3:**
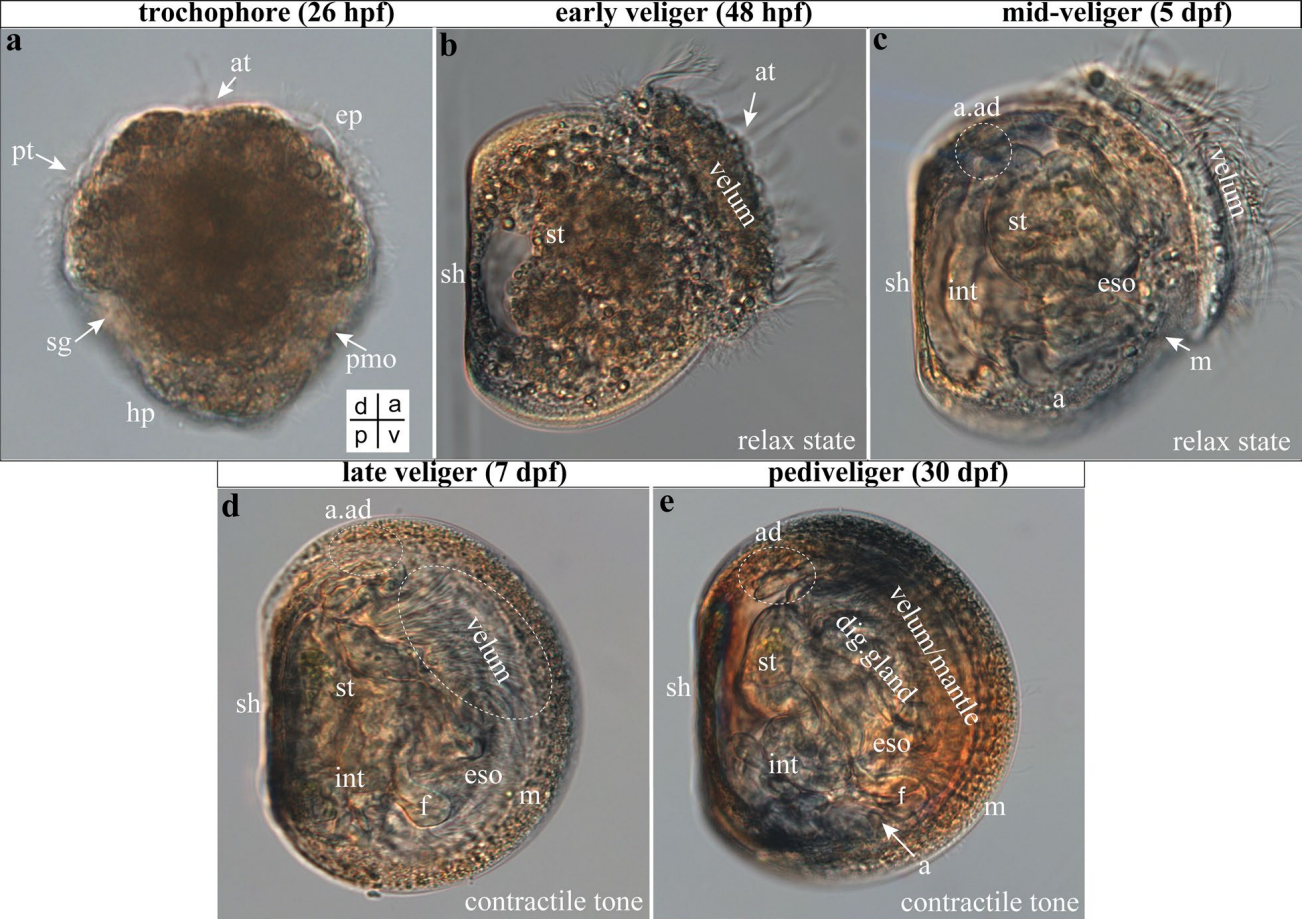
Some stages of development of the scallop *Azumapecten farreri*. Light microscopy images. **a** trochophore, **b** early veliger, **c** middle veliger, **d** late veliger, **e** pediveliger stage. Abbreviations: ad—anterior adductor, at—apical tuf, ep—episphere, eso—esophagus, hp—hyposphere, int—intestine, m—mouth, pmo—presumptive mouth opening, pt—prototroch, sg—shell gland, sh—shell, st—stomach, v—velum. The orientation a—anterior, d—dorsal, p—posterior, v—ventral. Scale bars = 20 μm

We monitored scallop development using light microscopy (Fig. 3), to select informative stages for immunofluorescence and histochemical analyses. After fertilization, the egg transforms into a ciliated blastula (not shown) that develops into a free-swimming trochophore by 26 h post fertilization (hpf) at 18 °C (Fig. 3a). The larva has an oval shape with an apical cilia tuft, cilia encircling the body (prototroch), and a presumptive mouth that is located beneath the prototroch on the ventral side and anlage of shell on the dorsal part of the trochophore (Fig. 3a). The veliger stage is D-shaped and characterized by a velum, a strongly ciliated lobe used for locomotion and gas exchange (Fig. 3b). The D-veliger and mid-veliger possess a well-defined shell and a developing digestive system (Fig. 3c, relaxed state). The late scallop veliger (7 dpf, contractile tone) has a well-developed digestive system, consisting of the mouth, esophagus, stomach, intestine, and anus. The larva retains the velum and becomes a visible foot (Fig. 3d). Pediveliger has a thick shell, more visible parts of the digestive system, and the cilia of the vellum is less visible than at the previous stage, which may indicate its resorption and development of the mantle (Fig. 3e).

### Nomenclature of the neuronal larval structures

The nomenclature and abbreviations of all neuronal structures in scallop larvae were used according to the suggested terms and definitions in a neuroanatomical glossary [21]. The names of neural processes were based on the ganglion they exit from and the ganglion they extend to (for example, cerebropleural connectives). Based on previous data on separate development of CPG [20, 22, 23], ganglia at the early scallop veliger stages are termed as cerebral and pleural ganglia. Additionally, names used for peripheral nerves combine the region of the ganglion from which the nerve extends or the tissue it innervates (for example, velum and esophageal neurons). The peripheral larval nervous system of scallops has not been identified in previous studies. Here we first described the enteric nervous system and named neurons and processes according to the organs where these neurostructures are located or according to their innervation targets.

#### 5-HT-lir larval structures

**Fig. 4:**
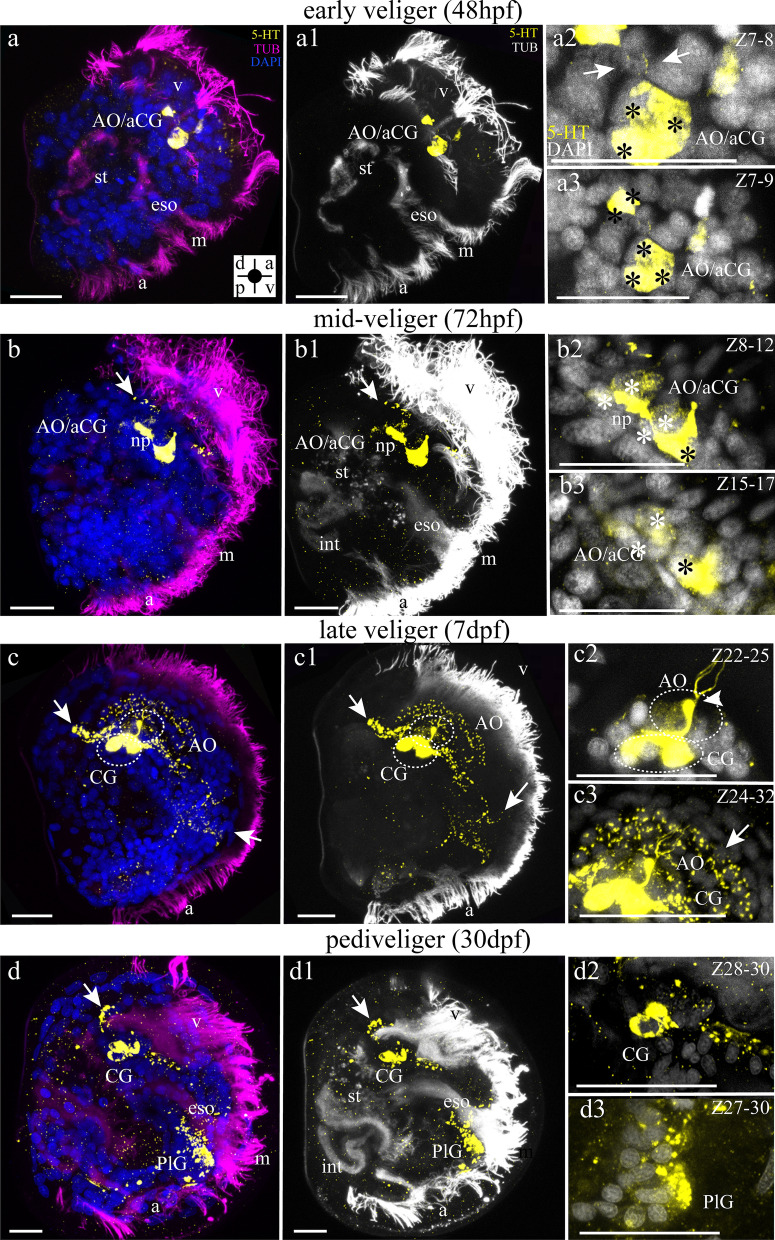
Serotonin immunoreactivity (5-HT-lir) in the veliger larvae of *Azumapecten farreri*. Yellow—5-HT-lir; mangeta (**a**–**d**) or white (**a1**–**d1**)—cilia, α-tubulin immunoreactivity; blue (**a**–**d**) or white (**a2**–**d2**)—cell nuclei, DAPI. **a** The early veliger at 48 hpf. The apical organ (AO) comprises five 5-HT-lir cells with thin neurites running to the velum (v). Inserts: **a1** Five cells with thin neurites (arrows). **a2** Five cells with thin neurites (arrow) extending to the velum. **b** The middle veliger stage at 72 hpf. Cells of the AO are surrounded by neuropil and extend neurites (arrows) to the velum. Inserts: **b1** Four paired cells of the AO (asterisks) and neurites extend to velum (arrow). **b2** The neuropil of the AO with extending neurites (arrow). **c** The late veliger stage at 7 dpf. The AO is transformed into a cerebral ganglion (CG), which has thin neurites extending to the velum. From CG, neurites extend to the dorsal (arrow) and ventral parts of the larvae (arrow). Likewise on the ventral side of the larvae, neurites form a net of an enteric nervous system (ens). Inserts: **c1** 5-HT-lir neurites extend from CG to the dorsal side of the larvae (arrow) and to the velum (arrow) and **c2** to the ventral side of the larvae and enteric nervous system (ens). **d** The pediveliger stage is 30 dpf. CG 5-HT-lir neurites extend to the dorsal part of larvae and to the ventral part of the larvae and enteric nervous system (ens). The two neurons located in the ventral part of the larvae are likely components of the prospective pleural ganglion (PlG). Inserts: **d1** 5-HT-lir innervation extends from CG to the dorsal side of the larva’ s body. **d2** 5-HT-lir cells like part of PlG. Additional abbreviations: a—anus, m—mouth, st—stomach. The orientation a- anterior, d—dorsal, p—posterior, v—ventral. The orientation a- anterior, d—dorsal, p—posterior, v—ventral. Scale bars = 20 μm

No serotonin-like immunoreactivity (5-HT-lir) was observed at the trochophore stage (Additional file 1: Fig. S1). The first detection 5-HT-lir sensory cells was found at an early veliger stage (48 hpf) (Fig. 4a). Three densely spaced 5-HT-lir cells with neurites were part of the developing non-paired sensory (ciliated) larval organ called the apical organ (AO) (Fig. 4a, a1, a2). At the mid-veliger stage (72 hpf), the number of 5-HT-lir cells increased and the morphology of some cell changes: two 5-HT-lir flask-shaped cells appear (Fig. 4b, b1, b2, b3). The dense plexus of cell processes, the neuropile, can be clearly identified between the cells (Fig. 4b, b1, b2). In late veligers (7 dpf), numerous 5-HT-lir neurites extend from the AO to innervate the velum and dorsoventral regions of the larva, including the mouth and anterior adductor (Fig. 4c, c1). At the late scallop veliger in addition to the epidermal AO, subepidermal anlage of the CG appear (Fig. 4c1–c3). Using 3D visualization, it is possible to distinguish the AO with cilia and the anlage of the CG in a seven-day-old veliger scallop (Additional file 2: Movie 1). The CG of pediveligers (30 dpf) develops by resorbing the AO, which results in the loss of sensory cilia (Fig. 4d, d1, d2). The CG neurites run to the anterior adductor and presumptive mantle. In pediveligers, a strong 5-HT signal appears in the precursor of pleural ganglia (PlG) and a weak signal in the anal region (Fig. 4d, d1, d3).

#### FMRFamide-lir larval structure

**Fig. 5:**
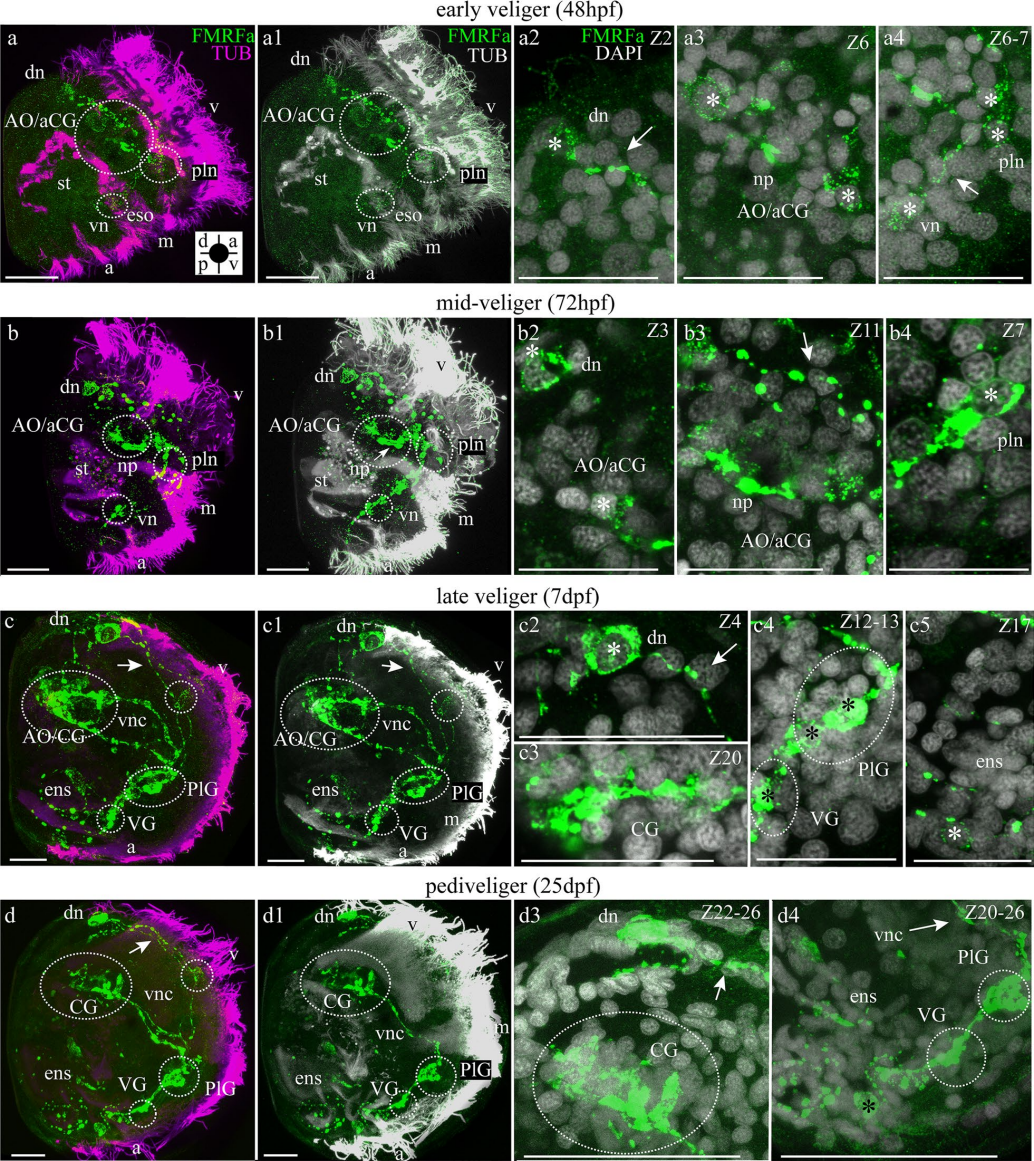
FMRFamide-like immunoreactivity (FMRFamide-lir) and acetylated α-tubulin staining and DAPI in the stages of the larvae of *Azumapecten farreri.* Green—FMRFamide, magenta—acetylated α-tubulin immunoreactivity (**a**–**d**) or white (**a1**–**d1**); white—nuclei, DAPI (**a2**, **a3**, **b2**, **b3**, **c2**–**c4**, **d3**, **d4**). The apical pole is always upward, and the ventral side is on the right. **a)** FMRFamide-lir components in the 48-hpf early veliger stage with apical organ (AO), dorsal neurons (dn) and neurites extends to the ventral part of the larvae (arrows). Inserts: **a1** Two paired cells of the apical organ (AO). **a2** Dorsal neurons (dn) with cilia (arrowhead) and extended to ventral side of the larvae FMRFamide-lir neurites (arrow). **b** The larva of the middle veliger stage on 72 hpf, dorsal neurons and the nerve cord extends from AO to the ventral side of the larvae–visceral nerve cord (vnc). Inserts: **b1** Number of cells in AO increased to eight (asterisks) and two paired dorsal neurons (asterisks, dn). **b2** The connection of the visceral nerve cord and nerve cord extending from the dorsal neurons on the ventral side of the larva with appeared FMRFamide-lir ventral neurons (vn). **b3** FMRFamide-lir neurites (arrow) in the ventral part of the larva and neurons of enteric innervation (asterisks). **c** Larvae at the veliger stage (7 dpf) demonstrate the presence of FMRFamide-lir elements in the CG, visceral ganglion (VG), pleural neurons (pln), dorsal neurons (dn) and enteric nervous system (ens). Inserts: **c1** Net of neurites (arrow) between dorsal neurons (asterisks), CG (asterisks). **c2, c3** Paired visceral nerve cords (vnc) connecting CG and pleural neurons (pln), which connect neurites (arrow) with VG and follows enteric innervation (arrows, asterisks, ens). **d** The 25-dpf pediveliger stage with the CG, paired visceral cords (vnc), pedal (PG), and visceral ganglia (VG) located along the visceraneural cord. Inserts: **d1** Dorsal neurons (asterisks, dn) with cilia (arrowhead), neurites (arrow) and cerebral ganglion CG. **d2** Visceral nerve cord (vnc) on the ventral side of the larva connected with pleural ganglion (PlG) and visceral ganglion (VG). **d3** FMFRa-lir neuron of the gut (asterisks, ens). Additional abbreviations: a—anus, m—mouth, st—stomach v—velum. The orientation a—anterior, d—dorsal, p—posterior, v—ventral. Scale bars = 20 μm

No FMRFamide-lir cells are observed at the trochophore stage (Additional file 1). FMRFamide-lir cells first appeared in several regions at once at an early veliger stage (48 hpf) (Fig. 5a, a1). Two FMRFamide-lir cells appear in the AO region and their neurites connect these cells (Fig. 5a2, a3). Additionally, FMRFamide-lir neurons emerged on the dorsal part of larva and their neurites were directed via AO to the ventral part of larva, where they connected with other paired FMRFamide-lir cells (pleural neurons) (Fig. 5a, a1–a4). At 72 hpf, the number of neuronal elements increased; the pleural neurons on the ventral side of larvae became clearly visible (Fig. 5b–b4). A nerve cord (visceral nerve cord) appeared between the AO, pleural, and ventral neurons (Fig. 5b, b1). FMRFamide-lir cells appeared on the ventral side of the larva (Fig. 5b, b1). By late veliger (7 dpf), larvae have a strongly stained AO/CG consisting of approximately eight FMRFamide-lir cells that are connected to dorsal neurons (Fig. 5c, c1, c2). Neurites of CG connect with dorsal neurons and pleural neurons. Pleural neurons, in turn, communicate with anlage of VG (Fig. 5c, c1, c4). The different neuronal structures are interconnected by neurites as summarized in Fig. 9.

We detected neuronal somata with neurites forming a ring around the intestine and neuronal somata in the anal region of late veliger (Fig. 5c, c1, c5). Pediveliger larvae (25 dpf) possessed a well-organized nervous system consisting of three ganglia (cerebral, pleural, and visceral) connected by connectives forming paired visceral nerve cords (Fig. 5d, d1, d2). Next to the paired CG, weak immunostaining of the cells of the resorbing AO can be observed (Fig. 5d, d1, d2). Paired dorsal neurons have cilia and paired long neurites running along the velum and are connected with nerves of the visceral cord that run to the pleural and VG (Fig. 5d–d3). A distinctive feature of the pediveliger is that it has a well-developed enteric nervous system, consisting of two anal FMRFamide-lir somata rings around the intestine and a couple FMRFamide-lir cells in the area of the stomach (Fig. 5d, d1, d3).

#### Mutual arrangement of FMRFamide-lir and 5-HT-lir larval structures

**Fig. 6:**
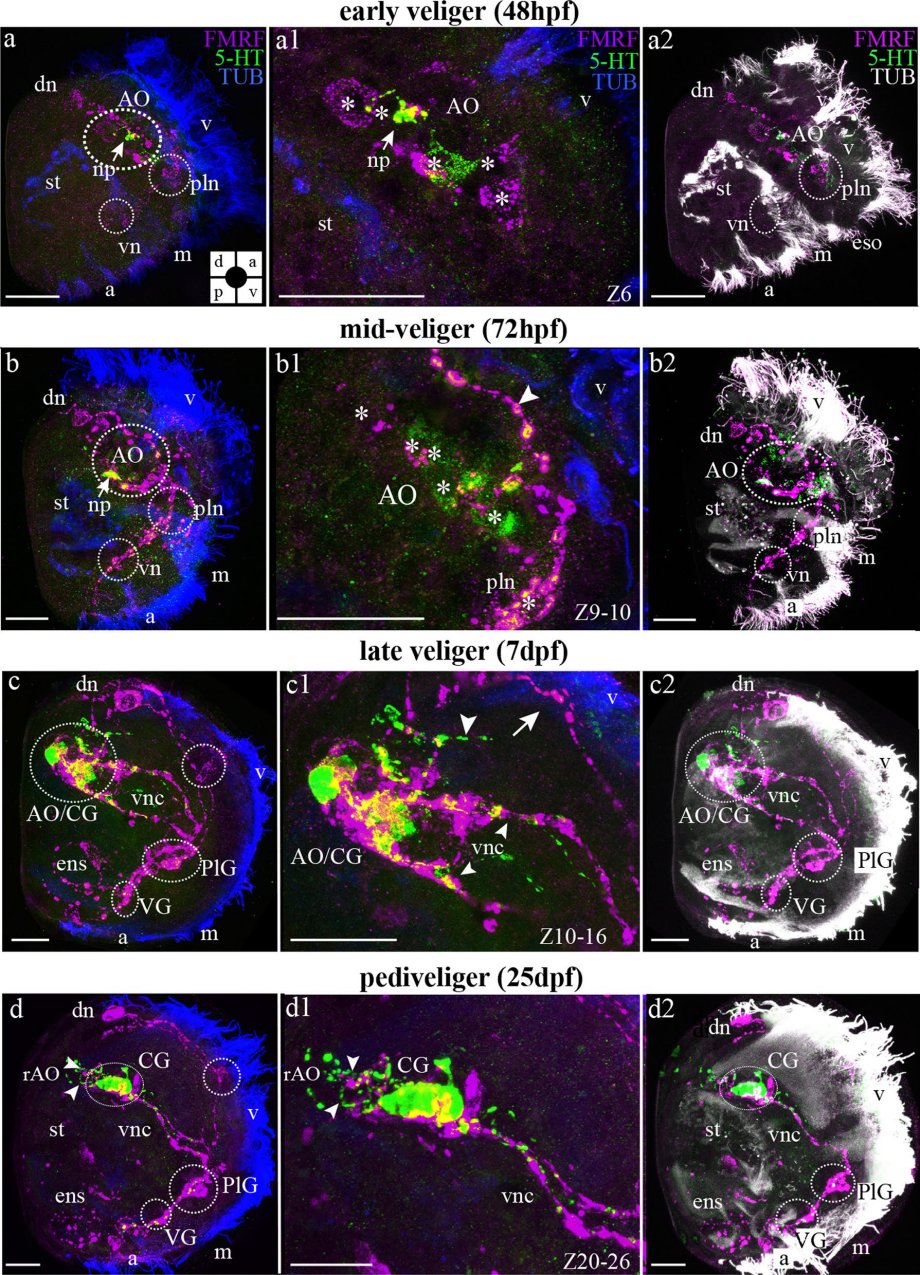
Mutual distribution of FMRFamide-lir, 5-HT-lir and acetylated α-tubulin in the stages of the larvae of *Azumapecten farreri*. Green: 5-HT-lir, magenta: FMRFamide-lir, blue (**a**–**d**, **a1**–**d1** or white (**a2**–**d2**)): acetylated α-tubulin-lir. **a** FMRFamide-lir and 5-HT-lir components of the larval nervous system in the 48-hpf early veliger stage with apical organ (AO), dorsal neuron (dn) and neurites extending to the ventral part of the larvae. Inserts: **a1** Two paired 5-HT-lir cells (asterisks) lie between two FMRFamide-lir cells (asterisk) of the apical organ (AO) near the apical tuft of cilia (at). In the central part of the AO, neurites form the neuropil. **b** The larva of the middle veliger stage is 72 hpf. AO includes 5-HT-lir cells (asterisk) and FMRFamidel-lir cells (asterisk). From FMRFamide-lir cells extending to the ventral side of the larva, there appear pleural neurons (pln) and down ventral neurons (vn). the dorsal part of the larva lie dorsal neurons (dn). The nerve cord extends from AO to the ventral side of the larvae—visceral nerve cord (vnc). Inserts: **b1** The AO including 5-HT-lir cells (asterisk) and FMRFamide-lir cells (asterisk). **c** Larva at the veliger stage (7 dpf) demonstrate the presence of FMRFamide-lir elements in the CG (transformed from AO) including 5-HT-lir cells and FMRFamide-lir cells, visceral ganglion (VG) include only FMRFamide-lir elements, pleural ganglion (PlG) include only FMRFamide-lir elements, FMRFamide-lir dorsal neurons (dn) and interstitial innervation (ems). Inserts: **c1** Transformed CG extending from four paired visceral cords (two 5-HT-lir cords and two FMRFamide-lir cords). **d** The 25-dpf pediveliger stage with the CG (including 5-HT-lir cells and FMRFamide-lir cells), four paired visceral cords (two 5-HT-lir cords and two FMRFamide-lir cords) (vnc), pedal ganglion (PG) including only FMRFamide-lir elements, and visceral ganglion (VG) including only FMRFamide-lir elements located along the visceral cords (vnc). Inserts: **d1** Cerebral ganglion CG and visceral cord. From CG to the dorsal part of the larvae extends 5-HT-lir neurites and FMRFamide-lir neurites. Additional abbreviations: a—anus, m—mouth, st—stomach v—velum. The orientation a—anterior, d—dorsal, p—posterior, v—ventral. Scale bars = 20 μm

Double-immunolabeling showed that FMRFamide and 5-HT antibodies detected different neurons and their neurites in early scallop veligers (48 hpf). FMRFamide-lir was detected in dorsal, pleural, and ventral neurons, as well as in the AO (Fig. 6a, a1, a2).. The 5-HT-lir apical neurons are located between the two FMRFamide-lir apical cells (Fig. 6a1). Dorsal FMRFamide-lir neurons project neurites towards ventral FMRFamide-lir cells that are not 5-HT-positive at this stage (Fig. 6a, a2). Later, in mid-veliger larva (72 hpf), the 5-HT-lir cells are located centrally within the AO and are surrounded by FMRFamide-lir cells (Fig. 6b, b1, b2). FMRFamide- and 5-HT-lir neurites were densely arranged in the neuropil of AO (Fig. 6b, b2). The colocalization of 5-HT and FMRFamide is visible in the neuropil, but this is not true colocalization (i.e. 5-HT and FMRFamide-lir in the not same cells/neurites) of the two markers and signals overlap due to close proximity of 5-HT/FMRFamide-lir structures (Fig. 6b, b1, b2). At this stage, the number of labeled somata/neurites increased and FMRFamide-lir was detected in dorsal, pleural, and ventral cells and their connectives (Fig. 6b, b2). In the late veliger, FMRFamide-lir cells are detected along the visceral nerve cords, throughout the enteric nervous system and within the cerebral, pleural, and VG. Furthermore, dorsal FMRFamide-lir neurons that extend neurites covering the velum region are still visible (Fig. 6c). At the same stage of development, 5-HT immunoreactivity is restricted to the CG and its neuropil (Fig. 6c, c1, c2). Later, in pediveliger, double labeling demonstrated the same situation of colocalization of FMRFamide-lir and 5-HT-lir in AO/CG and PlG (Fig. 6d, d1, d2).

#### Neuromorphology of the scallop larva during settlement

**Fig. 7:**
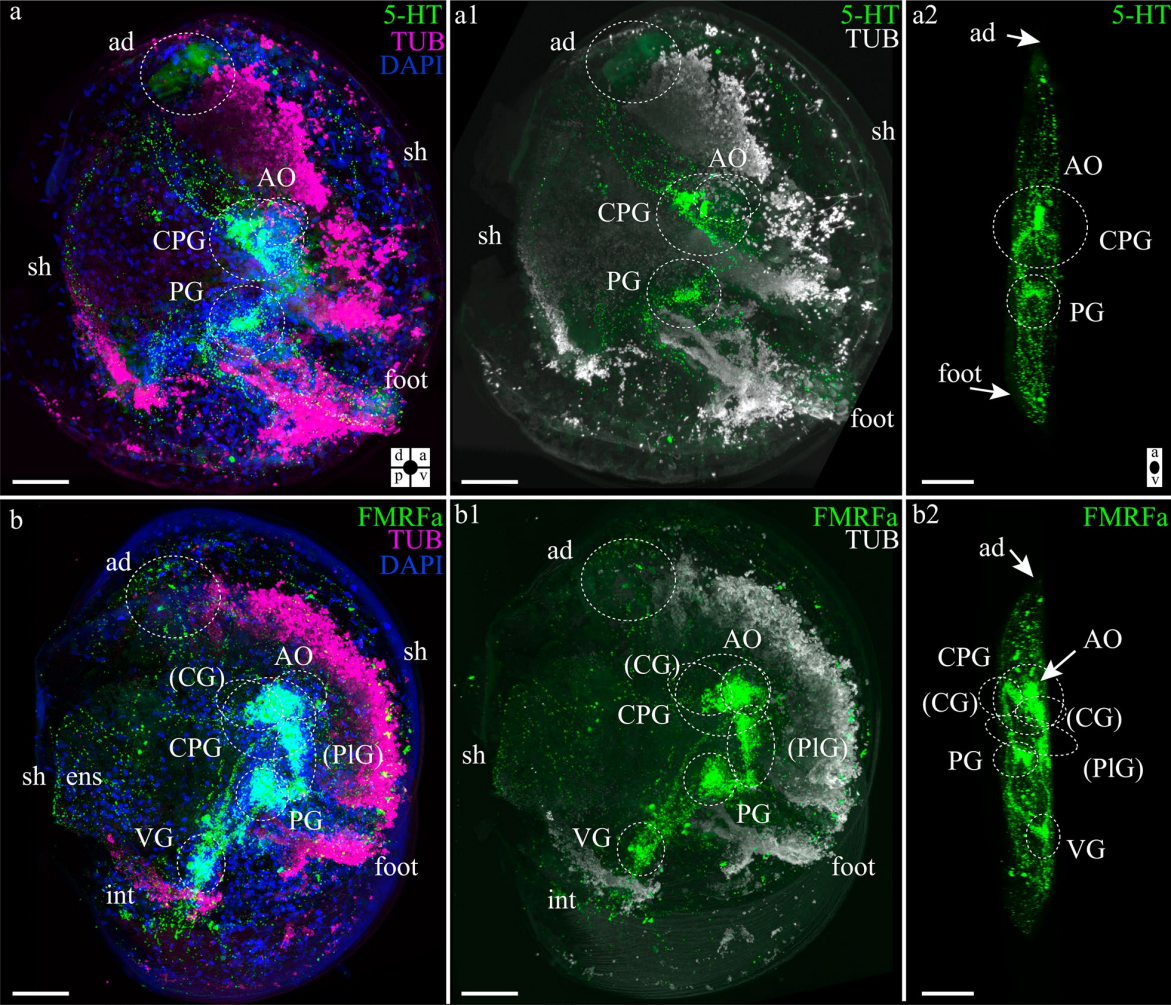
Neuromorphology of *Azumapecten farreri* pediveligers. **a** General view of 5-HT-lir (green) with the cerebropleural (CPG) and pedal (PG) ganglion together with acetylated α-tubulin (magenta) and DAPI (blue). **a1**–**a2** 5-HT-lir (green) innervation of the foot is visible. Acetylated α-tubulin (pseudo-color, white) reveals foot and resorbing velum. Peripheral innervations of the mantle, anterior adductor muscles (ad) and enteric nervous system (ens) are visible. **b** General view of FMRFamide-lir (green) with the cerebropleural ganglion (CPG), pedal (PG), and visceral (VG) ganglia, acetylated α-tubulin (magenta) and DAPI (blue). **b1**–**b2** The FMRFamide-lir (green) innervations of the mantle, anterior adductor muscles (ad), and enteric nervous system (ens). Acetylated α-tubulin (pseudo-color, white) reveals foot and resorbing velum. Additional abbreviations: int—intestine, st—stomach, sh—shell. The orientation a—anterior, d—dorsal, p—posterior, v—ventral. Scale bars = 50 μm

Late scallop larvae (55 dpf) have a complex nervous system. 5-HT immunostaining can be observed in the CPG, and the PG is located at the base of the foot and innervates it (Fig. 7a, a1, a2). The 5-HT-lir neurites of the CPG innervate the mantle, anterior adductor, and digestive system (Fig. 7a, a1). We did not find 5-HT-lir dense clusters of cells in the VG region, but thin neurites in the VG area were immunopositive for 5-HT (Fig. 7a, a1).

FMRFamide immunostaining showed distinctive features in the later stages which were (1) the convergence of the cerebral and pleural ganglia and (2) the formation of a single fused CPG (Fig. 7b, b1, b2). Despite the physical fusion of the ganglia, it was possible to distinguish between the cerebral (round-shaped) and pleural (elongated) parts of the ganglion (Fig. 7b, b1). The FMRFamide-immunoreactive system innervates the mantle and anterior adductor, and the FMRFamide-ergic neurites surrounds the stomach and intestines (Fig. 7b, b1). In the later stages of scallop development, FMRFamide became detectable in the PG (Fig. 7b, b1, b2) and was broadly distributed in the digestive system (Fig. 7b, b1).

Taken together, these data show that the ganglionic nervous systems of adult scallops is already prefigured at settlement, including an extensive innervation of the visceral organs.

#### Catecholamines development in scallop larvae

**Fig. 8:**
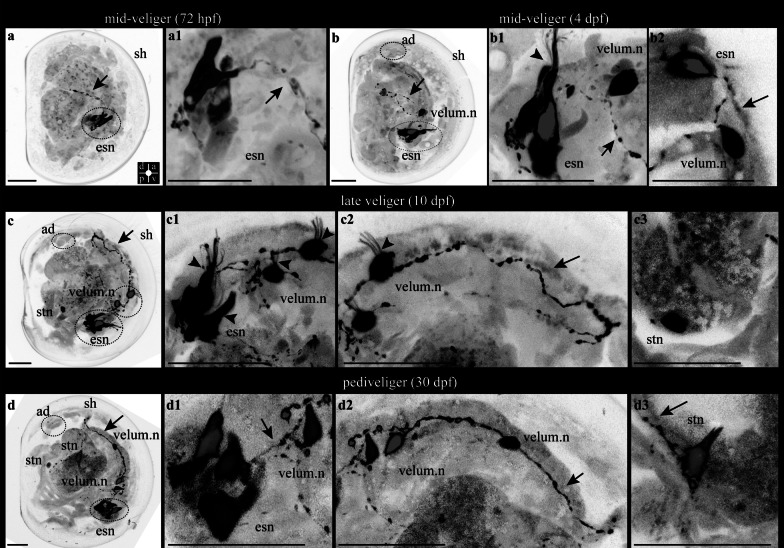
FaGlu fluorescence of the catecholaminergic nervous system of *A. farreri* larvae. **a** Larvae in the middle veliger stage in 72 hpf have two catecholaminergic paired esophageal neurons (asterisks, esn) in a pharyngeal zone with two paired neurites along the esophagus (arrow). Inserts: **a1** Magnified view of the two esophageal neurons (asterisks, esn) with neurites (arrow). **b** Middle veliger stage (4 dpf) has not only neurons of the esophagus (esophageal neurons, esn) and neurites but also innervation in the stomach of the larva like a net of neurites extending from esophageal neurons (arrows) and two appearing neurons in velum (velum.n). Inserts: **b1** Esophageal neurons (asterisks, esn) have many cilia (arrowheads) near two paired neurons of the velum (asterisks, velum.n) connected with esophageal neurons by neurites (arrow). **b2** The connection of the esophageal neurons with each other to neurites (arrows). **c** The 7 dpf-late veliger stage larva has esophageal neurons (esn, asterisk), velum.n with visible neurites along the velum (arrows), and more spread net of neurites with appeared catecholaminergic neuron (asterisks, stn) in the stomach zone. **c1** Increased number of esophageal neurons from two to four cells (asterisks, esn) with cilia (arrowheads) and velum’s neurons (velum, n) connected to each other and to esophageal neurons by catecholamine-positive neurites (arrow). **c2** Two neurons of the velum with extending catecholamine-positive nerve cord along the velum (arrow). **c3** Catecholamine-positive neuron in the stomach of the larva (asterisks, stn). **d** Pediveliger stage on 30 dpf has esophageal neurons (esn) with connecting neurites similar to that in previous stages, stomach neuron (stn) with a net-like innervation in the stomach zone, velum’ s neurons (velum, n) with a neuronal cord along the velum with appearing additional neurons in velum (asterisk, velum, n). Inserts: **d1** Six esophageal neurons of the pharyngeal zone (asterisks, esn), neurons of velum (asterisks, velum, n) and neurites (arrows). **d2** Nerve cord of the velum (arrows) with two pairs of neurons (asterisks, velum, n). **d3** Stomach innervation by two unpaired cells (asterisks, stn) with neurites (arrows). Additional abbreviations: v—velum. The orientation a—anterior, d—dorsal, p—posterior, v—ventral. Scale bars = 20 μm

The FaGlu fluorescence reaction is commonly employed to identify catecholamines [24] and is especially useful for non-immune detection of catecholamines (CA). No CA-positive structures were observed at the trochophore stage (Additional file 1). The CA-positive neurons were first detected at mid-veliger (starting from 72 hpf). Two flask-shaped cells adjacent to the esophagus near the larval mouth were found (Fig. 8a, a1). Neurites extended from each CA-ergic cell along the esophagus (Fig. 8a, a1). Later, the mid-veliger (4 dpf) had four esophageal CA-ergic neurons with numerous cilia and a common neuropil (Fig. 8b, b1, b2). At this stage of development, paired CA-ergic neurons appeared in the velum which were connected with esophageal neurons that spread their neurites to the stomach (Fig. 8b, b2). The late veliger (10 dpf) CA-ergic system is becoming more complicated: the group of sensory esophageal neurons (four neurons) is connected with multi-ciliary velum neurons, and their neurites form a neuronal ring in the velum (Fig. 8c, c1, c2). At the same time, other neurites of esophageal neurons connected with a newly appeared single stomach neuron (Fig. 8c, c3). Later, in pediveliger larvae (30 dpf), the number of sensory esophageal neurons increased to six, and their multiple cilia were not visible at this larval stage (Fig. 8d). Esophageal neurons connect with paired velum neurons by neurites extending through the entire velum (Fig. 8d, d1, d2). At this stage additional paired neurons appeared in the velum, which connected with paired neurons in the stomach along with other velum neurites (Fig. 8d, d3).

An additional pair of CA-positive neurons was observed in the velum of pediveligers, with neuronal connection to both CA-positive neurons in the stomach area and other velum neurons.

## Discussion

The nervous system of bivalves has a ganglionic (three ganglia: CPG, PG, and VG), tetraneural (paired lateral and visceral cord) structure [15, 17, 20, 25, 26]. In scallops, the structure of the nervous system differs from other bivalves, and this is reflected in the appearance of additional neurostructures, such as additional ganglia (Ag) and nerves associated with the innervation of the unique sensory structures of the scallop such as eyes, osphradium (osphradial nerves, branchial, pallial nerves) [8, 18, 27, 28].

We found that neurons containing peptide FMRFamide were detected in all three ganglia of an adult scallop, whereas 5-HT-lir neurons were found only in the accessory ganglion (Ag) adjacent to VG but 5-HT-lir neurites were identified in all ganglia. Our data on the nervous system of the scallop *A. farreri* are in agreement with the gross anatomy of the nervous system of other bivalve species [18, 20, 27, 29–34].

**Fig. 9:**
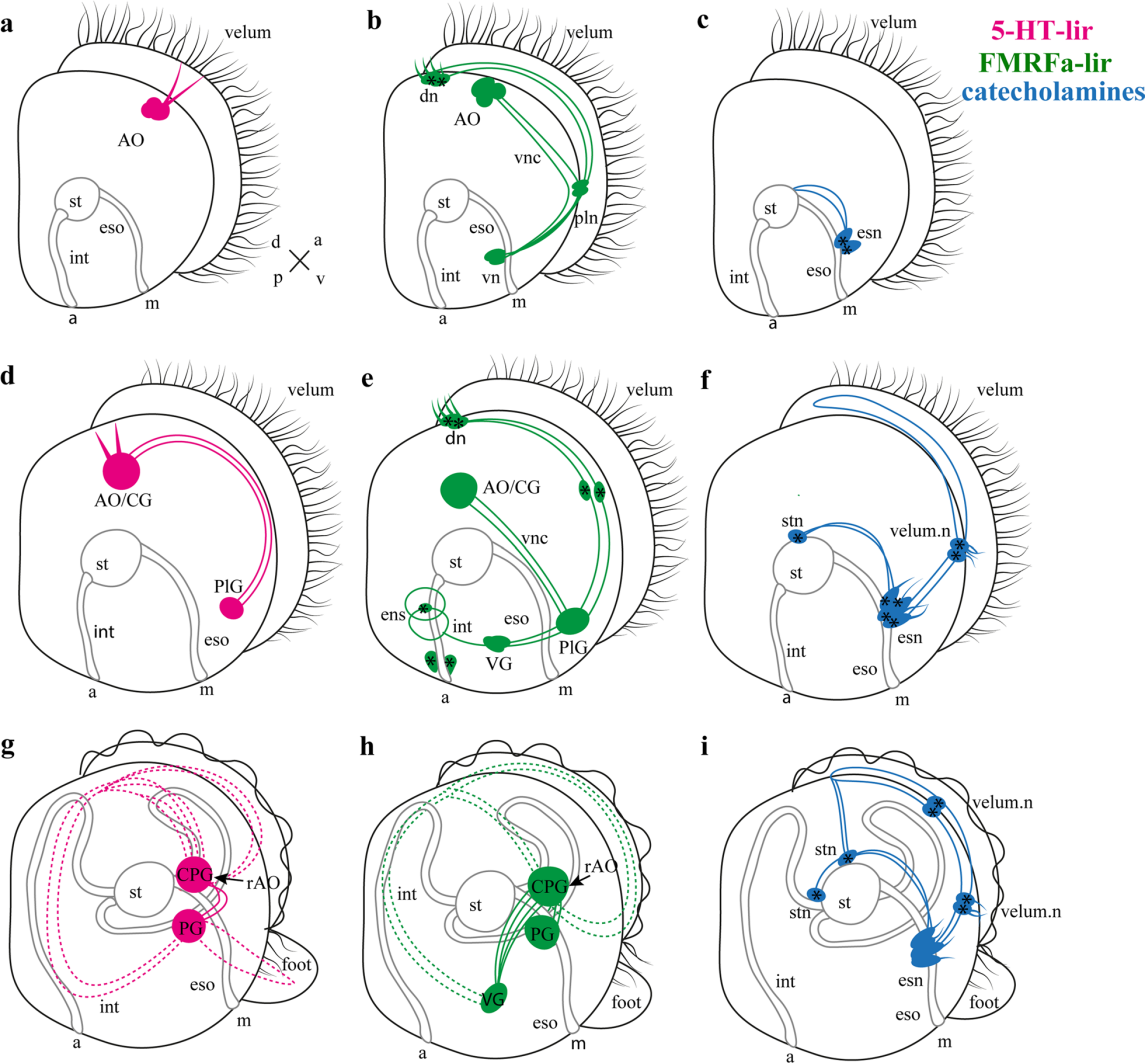
Schematic presentation of the neurogenesis of *A. farreri* larvae. Serotonin-immunoreactive (5-HT-lir) elements are magenta; FMRFamide-lir elements are green; and catecholaminergic elements are blue. Black outlines provide a schematic overview of larval morphology and organs. d/v and a/p—orientation of larvae on the scheme. Three developmental stages are shown: **a**–**c** early veliger, **d**–**f** late veliger, **g**–**i** pediveliger. Somata are shown as asterisks. Prospective ganglia are shown as colored circles with the corresponding signatures of the ganglia or AO. The colored lines represent neurites connecting neurons or ganglia. Dotted lines represent the innervation of the pediveliger organs. Abbreviations: a—anus, AO—apical organ, CG—cerebral ganglia, CPG—cerebropleural ganglion, dn—dorsal neurons, eso—esophagus, esn—esophagus neurons, int—intestine, m—mouth, PG—pedal ganglia, PlG—pleural ganglion, st—stomach, stn -stomach neurons, velum, n—neurons of the velum, VG—visceral ganglia, vn—ventral neurons, vnc—visceral nerve cords. The orientation a—anterior, d—dorsal, p—posterior, v—ventral

This study helped us understand the developmental history of the scallop nervous system, from the appearance of the first 5-HT-, FMRFamide-, and CA-positive cells to the transformation of the larva after settling (Fig. 9). We found that the first expressing neurotransmitter cells are 5-HT-lir cells and flask-shaped FMRFamide-lir cells, which matches data in the existing research of clams. These first immunopositive cells are part of the AO, which is a particularly well conserved larval sensory structure within Lophotrochozoa [22, 35–39].

However, the amount, priority, and morphology of neuronal cells appearing in the AO is taxon-specific for different among molluscan and bivalves taxa [22, 23, 39–42]. The quantitative and morphological differences of AO 5-HT-lir and FMRFamide-lir cells in early larvae of bivalves is shown in Table 1.

**Table 1:** AO composition in bivalve larvae

Species	5-HT and FMRF cells in AO of some early larval stages						References
	Late trochophore		Early veliger		Middle veliger		
	*5-HT-lir *	*FMRF-lir *	* 5-HT-lir *	*FMRF-lir *	*5-HT-lir *	*FMRF-lir *	
*Mytillus trossulus*	1 f-s	1 f-s	3 f-s	4-5 f-s	5 f-s	7 f-s	Voronezhskaya et al. [23]
*Crassostrea gigas*	2 f-s	0	3f-s +2r	2-4 f-s	5 r	1 f-s	Yurchenko et al. [22]
*Crassostrea gigas*	3 f-s	4 f-s	3f-s +2r	No data	No data	No data	Nezlin and Voronezhskaya [39]
*Spisula solidissima*	1 (CG/AG)	No data	3 (CG/AG)	No data	2 (CG/AG)	No data	Kreiling et al. [40]
*Dreissena polymorpha*	1 f-s	No data	3-4 f-s	No data	4 f-s+ 2r 2 f-s+ 4r 1f-s+5r	No data	Pavlicek et al. [41]
*Dreissena polymorpha*	1-2 f-s	1 f-s	2 f-s	2 f-s	3 f-s+ 2r	3f-s	Battonyai et al. [42]
*Azumapecten farreri*	0	0	3	2 f-s	5	4	Presented here

f-s, flask-shaped cells; r, round cells

For *A. farreri,* there are three 5-HT-lir cells between two FMRFamide-lir cells in the early veliger stage in AO (Fig. 9a, b). For example, in the veliger stage the *Crassostrea gigas* AO contains three 5-HT-lir flask-shaped cells and two rounded cells, while first neuronal cells appear on the trochophores stage [39]. The AO of *Mytilus trossulus* larvae in trochophores was found to have three 5-HT-lir flask-shaped cells and five 5-HT-lir cells later [23]. For the clam *Spisula solidissima,* the same number of flask-shaped 5-HT-lir cells was detected [40]. In *Dreissena polymorpha,* the number of 5-HT-lir flask-shaped cells of AO varies between two studies: one study demonstrated that the AO consists of three 5-HT-lir flask-shaped cells [41] but another described four 5-HT-lir flask-shaped cells in the AO [42].

All this data allows us to conclude that bivalve nervous system development generally commences with the formation of an AO. This first larval sensory structure initially comprises two to three 5-HT-lir and FMRFamide-lir cells, although the rate of its development varies from species to species.

The next important neurostructure is the visceral nerve cord. *A. farreri* visceral cord forms from neurites extending from AO/CG cells running to the ventral part of larvae (Fig. 9). Lengthwise to the visceral nerve cord structure, neurons appear, which are likely to be part of future ganglia. The first structure appearing along the visceral nerve cord is the pleural ganglion, which initially comprises only two paired FMRFamide-lir cells. Here we showed the independent formation of the pleural ganglion from pleural peripheral neurons, which subsequently converge with the cerebral ganglion, giving rise to the CPG of *A. farreri* (Fig. 9).

Early neurogenesis of scallop *A. farreri* resembles the neurogenesis described earlier in the oyster *C. gigas,* there are two first-appearing sensory FMRF-lir cells on the periphery in larvae, which will form ganglia. The role of peripheral neurons as characteristic for development of trochophore animals in the construction of the general plan of the nervous system is discussed in detail [22, 25]. Neurodevelopmental processes described in bivalves have 5-HT and FMRFamide-lir neurons of AO and FMRFamide-lir peripheral neurons in larvae [25].

Earlier studies of the development of the bivalve nervous system suggested that the AO cells are precursors of CG neurons [23, 43]. Consistent tracking of *A. farreri* developmental stages suggests that AO cells may be a part of developing CG in the late veliger stage by migration or displacement through morphogenetic movement. This suggestion is supported by data from *M. trossulus* and *D. polymorpha* neurogenesis representing continuity of ciliated flask-shaped cells of the AO into the CG by recent reconstructions of the serotoninergic nervous system [23, 42].

Formation of the PG for *A. farreri* larvae was detected as FMRFamide-lir and 5-HT-lir cells at the pediveliger stage at the same time as appearance of the foot [20, 28] (Fig. 9).

In late veligers of the oyster *C. gigas* and the mussel *M. trossulus*, PG neurons appear as rounded FMRFamide-lir and 5-HT-lir ventral neurons along each visceral cord in the region of the developing foot [23, 25]. *D. polymorpha* and *S. solidissima* did not have 5-HT- and FMRFamide-lir in PG at early developmental stages, but pediveliger larvae of these mollusks had a foot that exhibits activity [40, 42]. It is very likely that neurotransmitters appear in the PG of bivalves after the larvae settle.

In *A. farreri* larvae, ventral neurons appear in the early veliger stage as FRMFa-lir cells, which may become part of the future VG (Fig. 9). In *A. farreri* larvae, 5-HT-lir cells in the VG are absent as well as adult VG of scallop *A. farreri* (in the present study) and mussel *Crenomytilus grayanus* [15]. The first cells of VG larvae of the *M. trossulus* appear on the middle veliger stage (72 hpf) and do not contain 5-HT-lir cells but they are FRMFa-lir [23]. However, *C. gigas* VG contain FMRFamide-lir and 5-HT-lir neurons [22]. However, the question of whether these immunopositive cells are part of the definitive ganglia of bivalves remains to be answered.

The ventral FRMFamide-lir neurons of early *A. farreri* veligers may become part of the future VG (Fig. 9). This is consistent with data from *M. trossulus*, where the first VG neurons appear at the mid-veliger stage (72 hpf) and also contain FRMFamide [23]. Throughout all developmental and adult stages, 5-HT-lir somata are absent from the VG of the scallop *A. farreri* (present study) and the mussel *Crenomytilus grayanus* [15]. In contrast, the larval VG of *C. gigas* does contain FMRFamide-lir as well as 5-HT-lir neurons [22]. The question of whether larval neurons are persisted and whether they become part of the definitive ganglia of adult bivalves remains unclear.

The present study extends our knowledge of the presence of catecholamines in molluscs to the larval stages. The chromatographic data by Coon et al. (1986) [44], indicating the presence of catecholamines in bivalve larvae, and evidence from other studies suggest major roles for catecholamines in important functions such as locomotion, feeding, triggering of settling behavior, and metamorphosis [45, 46]. In *A. farreri,* the distribution of aldehyde-induced fluorescent cells demonstrated that CA-ergic neurons were located peripherally (Fig. 9). The first appearing CA-ergic cells at 3 dpf were similar to two flask-shaped neurons with cilia around the esophagus (Fig. 9). This suggests that CA regulates larval nutrition [47, 48]. Later, esophageal CA-positive cells forming the nerve cord extended down to the stomach (Fig. 9). At the same time, CA-ergic neurons emerged as velum innervation (Fig. 9). This is consistent with previous studies of the detection CA-ergic system in late veligers of scallop *Placopecten magellanicus* [49] and the mussel *M. edulis* [48]. Like the authors of this study, we did not find CA-ergic cells in the ganglia of the veliger of scallop *A. farreri*. Since CA neurons are present in the somata and neuropil of all major central ganglia of adult scallops *P. magellanicus* [49], it is very likely that these types of neurons in the ganglia appear after settling and metamorphosis, for example, in juvenile scallop individuals. In order to compare the cellular mechanisms of neurogenesis (the appearance of cells, their transmitter nature, and their quantitative assessment), as well as the future fate of cells before and after metamorphosis, further studies of later stages of development and using a wider range of species of bivalves are required.

## Conclusion

Comparative morphological analysis of *A. farreri* neurogenesis with other bivalves revealed common conserved characteristics, as well as distinctions in early neuronal specialization of the FMRFamide-lir and 5-HT-lir cells in veliger larvae. Based on data presented here, the larval nervous system of *A. farreri* consists of several (studied) subtypes of neurons (5-HT, FMRFamide-lir, CA-neurons) located in the early stages of larval development in different places (dorsal, ventral parts of larvae, esophagus region) with the exception of the AO, which includes 5-HT and FMRFamide-lir neurons. As the scallop late veliger larvae develop, they acquire features of centralization of the nervous system with the appearance of 5-HT and FMRFamide-lir ganglia and connections (connective) between them. The FMRFamide transmitter is widely distributed in the digestive system of late larvae, marking numerous somata and neurites along the intestine, while serotonin is sparsely represented in these locations. For the first time, the development of the CA-positive nervous system is described in detail for scallops and a conclusion is drawn about its sensory (or mechanosensory) role in larvae. We did not find CA cells in the ganglia, but all CA detected neurons were somehow connected with the locomotion organs and the digestive system, suggesting their participation in the innervation these organs and regulation of their physiological processes.

The nervous system of adult bivalves is decentralized (reduction of the head (cerebral) ganglia (in autobranch bivalves) or pedal ganglia in adults (oysters) due to a resorption of the foot or fusion (epiathroid state) ganglia (cerebropleural, autobranch bivalves) as compensator mechanism) [15, 20, 22, 23]. This is manifested in a decrease in CPG and a greater functional load on the VG of adult bivalves. In studied bivalve larvae, on the contrary: the nervous system of larvae consists of 5-HT and FMRFamide-lir neurons localized in developed CPG and PG, while VG is poorly developed and, in addition, there are no 5-HT neurons [present study, 8, 15]. Apparently, the mechanism of the decentralization of ganglia does not occur in larvae, but later, after their metamorphosis, which probably affects the reduction processes. Despite the fact that the VG takes over the functionality to ensure the work of visceral organs, the serotonin cells involved in many physiological processes are absent in it, and the serotonin center is represented by the CPG and PG in scallops as well as in mussel [15].

Summarizing the above data, the bivalve *A. farreri* possess morphology typical for bivalve ganglionic nervous systems and has, at the same time, a wide variety of neuronal cell types. These new results on the distribution of neurotransmitters in the adult CNS and larvae of *A. farreri* suggest that, although the nervous system of bivalves is secondarily reduced due to their sedentary lifestyle, their CNS neurons possess molecular heterogeneity to express neurotransmitters that are necessary for regulation of behavioral patterns.

## Methods

### Animals

Adults of *Azumapecten farreri* were collected in May–June 2019–2020 at the Vostok marine station of the A.V. Zhirmunsky National Scientific Center of Marine Biology (42°53′36.8" N, 132°44′03.1" E,10-m depth), an estuary on the inner part of the Vostok Bay and the Peter the Great Bay of the Sea of Japan. These animals were maintained in running seawater with aeration before performing experiments with those which were 6–10 cm long, 6–8 cm wide, and 3–4 cm thick.

### External morphological features for species identification

*A. farreri* shell is orbicular, equilateral, scaly, and the left valve is somewhat more convex than the right, the former bearing 10 coarse costae, and the latter about 25, between which, on both valves, appear numerous finer riblets; the auriculae are very unequal, with a large anterior, descending in a curve, while the posterior is comparatively small, forming approximately an obtuse-angled triangle, but sculptured with scaly riblets; the color is color dirty white, banded, and mottled with rich purple brown, especially on the left valve.

### Obtaining the histological material of the nervous tissue of adults

Samples of the ganglia of each specimen (cerebro-pleural, pedal, and visceral) were collected using a scalpel, incising the trailing muscles to open the shells of the animals. Immediately after extraction, the samples were placed in a 4% paraformaldehyde (PFA) solution, in a ratio of 0.5 cm^3^ of tissue to 5 mL of solution for 2–3 h at room temperature (20–22 °C). Then, the samples were washed with phosphate-buffered saline (PBS; 3 × 20 min). The material of each ganglion was transferred to a 30% sucrose solution and left for incubation overnight at 4 °C. The samples were embedded in a freezing medium (OCT cryomount, HistoLab, Finland) and frozen for storage at -20 °C. Tissue cryosections were then obtained using a cryostat HM525 (Thermo Fisher Scientific) with a thickness of 14 μm.

### Immunohistochemistry of adult animal ganglia

Before staining, the samples were rinsed with PBS with 0.1% Tween 20 (PBST) for 30 min to remove the freezing medium. The tissue materials on the glass slides were incubated overnight in a blocking solution (10% donkey normal serum, 1% bovine serum albumin, and 1% Triton X-100, 0.003% NaN_3_ in 0.1 M PBS) at 4 °C. The next step was the incubation of primary antibodies (Abs): serotonin goat polyclonal Abs (ImmunoStar, 20079) with FMRFamide rabbit polyclonal Abs (ImmunoStar, 20091) and with monoclonal mouse acetylated α-tubulin in the blocking solution at a dilution of 1:1000 for overnight at 4 °C. The samples were washed (5 × 20 min) in PBS and placed overnight in the blocking solution and then incubated for two hours at room temperature in secondary antibodies (Alexa Fluor 555 donkey anti-goat IgG (DAG) (Invitrogen, A32816), Alexa Fluor 488 donkey anti-rabbit IgG (DAR) (Invitrogen, A32814), Alexa Fluor 647 donkey anti-mouse IgG (DAM) (Invitrogen, A32787)) with a dilution of 1:1000 and 0.1 μg/mL DAPI. Further, the tissue was washed in PBST (5 × 20 min). A drop of 70% glycerol was added to the specimens on the glass slides and then they were covered by coverslips. As a control for non-specific immunorecognition, we performed immunohistochemical staining without the primary antibodies, adding only the secondary antibodies or normal (non-immunized) immunoglobulin G (1:500–1:1000; Sigma-Aldrich; I5006, I5381, and I5256).

### Larval culture

Male and female *A. farreri* scallops were submerged in different reservoirs with aerated seawater at 10–12 °C. Each animal was subjected to heat shock to obtain the gametes [4, 43]. The received larvae were cultured in beakers containing approximately 5 L filtered seawater with constant agitation of the water by an air jet directed at the water surface. The water was changed every 2 days. Starting from 24 h post-fertilization (hpf), the larvae were fed microalgae (100,000 cells/mL) [22]. During the first five days of larval development, the samples were fixed every 24 h after fertilization. Subsequently, the larvae were fixed at 7, 10, 15, 20, 25, 30 and 55 days of post-fertilization. A 7.5% MgCl_2_ solution was added to the larval suspension for anesthesia. The larvae were fixed in 4% PFA solution in phosphate buffer (PBS; 100 mM Na_3_PO_4_, 140 mM NaCl, pH 7.4) for 2–3 h at room temperature [22]. The fixed larvae were washed in 0.1 M PBS. The samples were dehydrated using methanol solutions in increasing concentrations (25%, 50%, 75%, and 100%) and stored in 100% methanol at − 20 °C.

The development of the larval culture was performed at 18 °C in order to avoid heterogeneity of the larvae. Each stage of scallop development was determined based on morphological, morphometrical, and behavioral features examined at each 24-h interval throughout all larval stages. Since the later stages of development of the scallop were heterogeneous we selected the larvae for immunocytochemistry only by close morphometric parameters of the shell.

### Immunocytochemistry of larvae

We used a previously described whole-mount immunostaining protocol [22, 50]. The larvae in each developmental stage in 100% methanol were transferred to 0.1 M PBS, by changing the solutions with decreasing concentrations of methanol. The samples were incubated overnight in 1% ethylenediaminetetraacetic acid (EDTA) in PBS at room temperature for decalcification. The samples were rinsed in PBS supplemented with 0.1% Triton X-100 (PBST) for 4 × 30 min, with agitation. Then, the samples were incubated overnight in a blocking solution (10% donkey normal serum, 1% bovine serum albumin, and 1% Triton X-100, 0.003% NaN_3_ in 0.1 M PBS For detection of the nerve structure, the larvae were incubated with primary antibodies at 4 °C in several combinations: (1) serotonin rabbit Abs (ImmunoStar, 20080) with monoclonal mouse antibodies raised against acetylated α-tubulin (ThermoFisher, RM318) (Figs. 4, 7); (2) FMRFamide rabbit polyclonal Abs (ImmunoStar, 20091) with monoclonal mouse acetylated α-tubulin (Figs. 5, 7); (3) Serotonin goat polyclonal Abs (ImmunoStar, 20079) with FMRFamide rabbit polyclonal Abs (ImmunoStar, 20091) with monoclonal mouse acetylated α-tubulin (Fig. 6) in the blocking solution at a dilution of 1:1000 for 5 days at 4 °C. Then, after washing in PBS (4 × 20 min) the samples were incubated overnight at 4 °C in donkey anti-goat (Invitrogen, A32814), donkey anti-rabbit (Invitrogen A32794), and donkey anti-mouse (Invitrogen, A32787) antibodies at a dilution of 1:1000 with 0.1 μg/mL DAPI. The larvae were then washed in PBST (5 × 20 min). All specimens were prepared for confocal microscopy and mounted on glass slides in a drop of 70% glycerol.

For controls, we showed that pre-incubation of the 5-HT antibody with the same conjugate (10 μg/mL, ImmunoStar, Cat. No. 20081) at 4 °C overnight eliminated all immunolabeling of serotonin in the tissues. The preadsorption of the diluted rabbit/goat antiserum with 10 mg/mL bovine serine albumin (BSA) overnight at 4 °C did not influence this staining, i.e., these antibodies recognized only serotonin and not BSA (Additional file 1). Also, only secondary antibodies (without treatment of primary antibodies) were used for control antibodies on veliger larvae (Additional file 1). Not fewer than 100 early, middle, and late veligers and 20–30 pediveligers were examined at each stage with each antibody. The numbers of immunopositive cells were counted at least 70% Abs stained larvae. Only cells with a visible nucleus were counted.

### Formaldehyde-glutaraldehyde-induced fluorescence (FaGlu) of larvae

For the detection of catecholamines in scallop larvae, we used formaldehyde-glutaraldehyde-induced fluorescence technique (FaGlu) as described earlier [24]. In aquatic animal models, a mixture of aldehydes forms stable fluorescent products with some catecholamines (adrenaline, noradrenaline, dopamine, dopa, 5-hydroxytryptamine and 5-hydroxytryptophan, but not with histamine or octopamine) and therefore can be easily visualized in the bodies of neurons and their neurites [51]. Scallop larvae (starting with the trochophore) were fixed for 4 h at room temperature in 4% PFA/0.5% glutaraldehyde and 30% sucrose in 0.1 M PBS (pH 7.4) and then rinsed with 0.1 M PBS, air-dried overnight, and mounted between glass coverslips using Mowiol medium (Calbiochem, San Diego, CA, USA). For negative control, different fixations, 4% PFA fixation and 0.5% glutaraldehyde separately were used.

### Confocal microscopy

Samples of larvae stained by immunocytochemical staining and histological sections of ganglia were scanned using an LSM 780 confocal microscope (Zeiss, Germany) and Zen software using lasers with the following wavelengths: 405, 488, 555, and 647 nm. All images of the larvae were prepared in the Z-stack mode with an optical slice thickness of 1 μm along the Z axis. The obtained images were transformed into projections at maximum intensity mode. All obtained images were analyzed using the ImageJ software (USA) [52].

For the detection of FaGlu fluorescence, we used a Zeiss LSM 780 laser-scanning microscope (Carl Zeiss, Oberkochen, Germany), operated in λ-mode. The excitation wavelength was 405 nm and the emission signal registered at 32 evenly spaced wavelengths (8.9 nm apart) from 408 to 693 nm using a QUASAR detector. FaGlu fluorescence in larvae was positive in the range 475–520 nm but not in ranges of 408–450 and 530–693 nm (Additional file 1). The results obtained were unmixed linearly using the Zeiss Zen 2.1 SP3 (Black Edition) software.

## Supplementary Information

**Additional file 1: Fig. S1.** Controls. Adult ganglia: immunostaining by secondary DAM, DAR, DAG antibody only (**A**–**C**). Larvae: detection of neurotransmitters 5-HT (**D**) and FMRFamide (**E**) in trochophores. Specificity of antibodies (**F**–**H**). Immunostaining by 5-HT after preadsorption of serum with serotonin (**F**) and immunostaining by secondary antibody only (**G**, **H**). FaGlu fluorescence in trochophores (**I**) and controls of FaGlu: 408–450 nm range (**J**) and 530–693 nm range ( K).

**Additional file 2: Movie 1.** 3D visualization of the AO with cilia and the anlage of the CG in a seven-day-old veliger scallop. The larva is immunostained with serotonin antibodies. Yellow is 5-HT-lir.

## Data Availability

Not applicable.

## Abbreviations

a: Anus
ad: Anterior adductor muscles
Ag: Accessory ganglion
AO: Apical organ
at: Apical tuf
CG: Cerebral ganglia
CPG: Cerebropleural ganglion
cppc: Cerebropleuro-pedal connective
cpvc: Cerebropleuro-visceral connective
dc lobe: Dorsal central lobe
dn: Dorsal neurons
ens: Enteric nervous system
ep: Episphere
eso: Esophagus
esn: Esophagus neurons
f-s: Flask-shaped cells
hp: Hyposphere
int: Intestine
lat.lobe: Lateral lobes
m: Mouth
n: Neurites
np: Neuropile
PG: Pedal ganglia
PlG: Pleural ganglion
pln: Pleural neurons
pmo: Presumptive mouth opening
pn: Pallial nerves
pt: Prototroch
r: Rounded cells
sg: Shell gland
sh: Shell
st: Stomach
stn: Stomach neurons
vc lobe: Ventral central lobe
velum.n: Neurons of the velum
VG: Visceral ganglia
vn: Ventral neurons
vnc: Visceral nerve cords

## References

[1] Wilkens LA, Ache BW. Visual responses in the central nervous system of the scallop. *Pecten ziczac. Experientia.* 1977; 33:1338–1340 https://books.google.de/books?id=vhGdBAAAQBAJ&dq=wilkens+ache+1977&source=gbs_navlinks_s

[2] Odintsova N, Dyachuk V, Kiselev K, Shelud’ko N (2006). Expression of thick filament proteins during ontogenesis of the mussel *Mytilus trossulus* (Mollusca: Bivalvia). Comput. Biochem. Physiol. B Biochem. Mol. Biol..

[3] Odintsova N, Dyachuk V, Nezlin L (2010). Muscle and neuronal differentiation in primary cell culture of larval *Mytilus trossulus* (Mollusca: Bivalvia). Cell Tissue Res.

[4] Dyachuk V, Wanninger A, Voronezhskaya EE (2012). Innervation of bivalve larval catch muscles by serotonergic and FMRFamidergic neurons. Acta Biol Hung.

[5] Leprêtre M, Almunia C, Armengaud J, Salvador A, Geffard A, Palos-Ladeiro MJ (2019). The immune system of the freshwater zebra mussel, Dreissena polymorpha, decrypted by proteogenomics of hemocytes and plasma compartments. Proteomics.

[6] Dyachuk V (2016). Hematopoiesis in Bivalvia larvae: Cellular origin, differentiation of hemocytes, and neoplasia. Dev Comp Immunol.

[7] Schmidt-Rhaesa A, Harzsch S, Purschke G (2016). Structure and evolution of invertebrate nervous systems.

[8] Kotsyuba E, Dyachuk V (2022). Effect of air exposure-induced hypoxia on neurotransmitters and neurotransmission enzymes in ganglia of the scallop *Azumapecten farreri*. Int J Mol Sci.

[9] Gillette R (2006). Evolution and function in serotonergic systems. Integr Comp Biol.

[10] Moncada S, Higgs EA (1991). Endogenous nitric oxide: physiology, pathology and clinical relevance. Eur J Clin Invest.

[11] Dockray GJ (2004). The expanding family of -RFamide peptides and their effects on feeding behaviour. Exp Physiol.

[12] Bechtold DA, Luckman SM (2007). The role of RFamide peptides in feeding. J Endocrinol.

[13] Mousley A, Novozhilova E, Kimber MJ, Day TA (2010). Neuropeptide physiology in helminths. Adv Exp Med Biol.

[14] Zatylny-Gaudin C, Favrel P (2014). Diversity of the RFamide peptide family in mollusks. Front Endocrinol.

[15] Kotsyuba E, Kalachev A, Kameneva P, Dyachuk V (2020). Distribution of molecules related to neurotransmission in the nervous system of the mussel *Crenomytilus grayanus*. Front Neuroanat.

[16] Vekhova E, Ivashkin E, Yurchenko O, Chaban A, Dyachuk V, Khabarova M, Voronezhskaya E (2012). Modulation of *Mytilus trossulus* (Bivalvia: Mollusca) larval survival and growth in culture. Acta Biol Hung.

[17] Dakin WJ (1926). The eyes of pecten, spondylus, amussium and allied lamellibranchs, with a short discussion on their evolution. Proc R Soc Lond.

[18] Matsutani T, Nomura T (1986). Serotonin-like immunoreactivity in the central nervous system and gonad of the scallop, *Patinopecten yessoensis*. Cell Tissue Res.

[19] Paulet YM, Dorval A, Benkhadra F (1993). Monoamines and reproduction in Pecten maximus, a preliminary approach. Invertebr Reprod Dev.

[20] Bullock TH, Horridge GA (1965). Structure and function in the nervous system of invertebrates.

[21] Richter S, Loesel R, Purschke G, Schmidt-Rhaesa A, Scholtz G, Stach T, Harzsch S (2010). Invertebrate neurophylogeny: suggested terms and definitions for a neuroanatomical glossary. Front Zool.

[22] Yurchenko OV, Skiteva OI, Voronezhskaya EE, Dyachuk VA (2018). Nervous system development in the Pacific oyster, *Crassostrea gigas* (Mollusca: Bivalvia). Front Zool.

[23] Voronezhskaya EE, Nezlin LP, Odintsova NA, Plummer JT, Croll RP (2008). Neuronal development in larval mussel *Mytilus trossulus* (Mollusca; Bivalvia). Zoomorphology.

[24] Furness JB, Costa M, Wilson AJ (1977). Water-stable fluorophores, produced by reaction with aldehyde solutions, for the histochemical localization of catechol- and indolethylamines. Histochemistry.

[25] Yurchenko OV, Savelieva AV, Kolotuchina NK, Voronezhskaya EE, Dyachuk VA (2019). Peripheral sensory neurons govern development of the nervous system in bivalve larvae. EvoDevo.

[26] Wanninger A (2009). Shaping the things to come: ontogeny of lophotrochozoan neuromuscular systems and the tetraneuralia concept. Biol Bull.

[27] Too CK, Croll RP (1995). Detection of FMRFamide-like immunoreactivities in the sea scallop *Placopecten magellanicus* by immunohistochemistry and western blot analysis. Cell Tissue Res.

[28] Daniel I, Speiser L, Wilkens A (2016). Neurobiology and behaviour of the scallop. Scallops: biology, ecology, aquaculture, and fisheries.

[29] Tantiwisawaruji S, Rocha E, Kovitvadhi U, Rocha MJ (2014). The bivalve nervous system and its relevance for the physiology of reproduction. Indian J Anat.

[30] Meechonkit P, Kovitvadhi U, Chatchavalvanich K, Sretarugsa P, Weerachatyanukul W (2010). Localization of serotonin in neuronal ganglia of the freshwater pearl mussel, *Hyriopsis (Hyriopsis) bialata*. J Molluscan Stud.

[31] Siniscalchi A, Cavallini S, Sonetti D, Sbrenna G, Capuano S, Barbin L, Turolla E, Rossi R (2004). Serotonergic neurotransmission in the bivalve *Venus verrucosa* (Veneridae): a neurochemical and immunohistochemical study of the visceral ganglion and gonads. Mar Biol.

[32] Karhunen T, Airaksinen MS, Tuomisto L, Panula P (1993). Neurotransmitters in the nervous system of *Macoma balthica* (Bivalvia). J Comp Neurol.

[33] De Biasi S, Vitellaro-Zuccarello L (1987). Distribution of 5-HT-immunoreactivity in the pedal ganglion of *Mytilus galloprovincialis*. Cell Tissue Res.

[34] De Biasi S, Vitellaro-Zuccarello L, Blum I (1984). Histochemical localization of monoamines and cholinesterases in Mytilus pedal ganglion. Histochemistry.

[35] Temereva E, Wanninger A (2012). Development of the nervous system in *Phoronopsis harmeri* (Lophotrochozoa, Phoronida) reveals both deuterostome- and trochozoan-like features. BMC Evol Biol.

[36] Wanninger A (2008). Comparative lophotrochozoan neurogenesis and larval neuroanatomy: recent advances from previously neglected taxa. Acta Biol Hung.

[37] Hay-Schmidt A (2000). The evolution of the serotonergic nervous system. Proc R Soc L.

[38] Croll RP, Voronezhskaya EE (1995). Early FMRFamide-like immunoreactive cells in gastropod neurogenesis. Acta Biol Hung.

[39] Nezlin LP, Voronezhskaya EE (2017). Early peripheral sensory neurons in the development of trochozoan animals. Ontogenez.

[40] Kreiling JA, Jessen-Eller K, Miller J, Seegal RF, Reinisch CL (2001). Early development of the serotonergic and dopaminergic nervous system in *Spisula solidissima* (surf clam) larvae. Comp Biochem Physiol A Mol Integr Physiol.

[41] Pavlicek A, Schwaha T, Wanninger A (2018). Towards a ground pattern reconstruction of bivalve nervous systems: neurogenesis in the zebra mussel *Dreissena polymorpha*. Org Divers Evol.

[42] Battonyai I, Voronezhskaya EE, Obukhova A, Horváth R, Nezlin LP, Elekes K (2018). Neuronal development in the larvae of the invasive biofouler *Dreissena polymorpha* (Mollusca: Bivalvia), with special attention to sensory elements and swimming behavior. Biol Bull.

[43] Dyachuk V, Odintsova N (2009). Development of the larval muscle system in the mussel *Mytilus trossulus* (Mollusca, Bivalvia). Dev Growth Differ.

[44] Coon SL, Bonar DB, Weiner RM (1986). Chemical production of cultchless oyster spat using epinephrine and norepinephrine. Aquaculture.

[45] Beiras R, Widdows J. Induction of metamorphosis in larvae of the oyster *Crassostrea gigas* using neuroactive compounds. *Marine Biology*.1995;327–334

[46] Bonar DB, Coon SL, Walch M, Weiner RM, Fitt W (1990). Control of settlement and metamorphosis by endogenous and exogenous chemical cues. Bull Mar Sci.

[47] Voronezhskaya EE, Hiripi L, Elekes K, Croll RP (1999). Development of catecholaminergic neurons in the pond snail, *Lymnaea stagnalis*: I. Embryonic development of dopamine-containing neurons and dopamine-dependent behaviors. J Comp Neurol.

[48] Croll RP, Jackson DL, Voronezhskaya EE (1997). Catecholamine-containing cells in larval and postlarval bivalve molluscs. Biol Bull.

[49] Smith SA, Nason J, Croll RP (1998). Distribution of catecholamines in the sea scallop, *Placopecten magellanicus*. Can J Zool.

[50] Dyachuk V, Odintsova N (2013). Larval myogenesis in Echinodermata: conserved features and morphological diversity between class-specific larval forms of Echinoidae, Asteroidea, and Holothuroidea. Evol Dev.

[51] Wreford NGM, Singhaniyom W, Smith GC (1982). Microspectrofluorometric characterization of the fluorescent derivatives of biogenic amines produced by aqueous aldehyde (Faglu) fixation. Histochem J.

[52] Schneider CA, Rasband WS, Eliceiri KW (2012). NIH Image to ImageJ: 25 years of image analysis. Nat Methods.

