# Supplementary figures and images for: Neurogenesis of the scallop Azumapecten farreri: from the first larval sensory neurons to the definitive nervous system of juveniles
Source: Front Zool. 2022 Aug 3;19:22. doi: 10.1186/s12983-022-00468-7 (PMC9347173; doi:10.1186/s12983-022-00468-7)

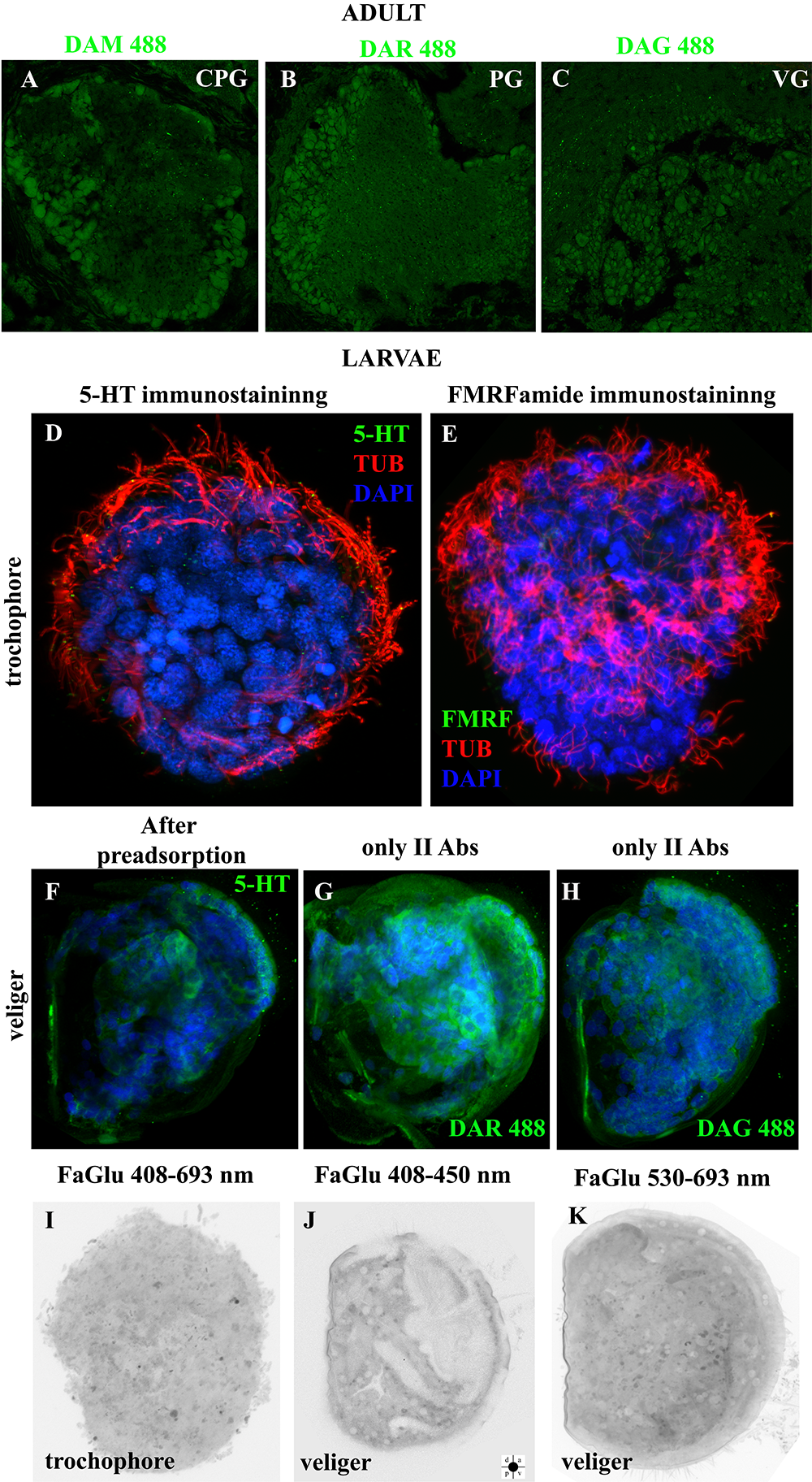

Supplement: Supplementary file 1 — Additional file 1: Fig. S1. Controls. Adult ganglia: immunostaining by secondary DAM, DAR, DAG antibody only (A–C). Larvae: detection of neurotransmitters 5-HT (D) and FMRFamide (E) in trochophores. Specificity of antibodies (F–H). Immunostaining by 5-HT after preadsorption of serum with serotonin (F) and immunostaining by secondary antibody only (G, H). FaGlu fluorescence in trochophores (I) and controls of FaGlu: 408–450 nm range (J) and 530–693 nm range ( K). [file 12983_2022_468_MOESM1_ESM.tif]
